# Benign but bizarre: A case report on idiopathic scrotal calcinosis

**DOI:** 10.1016/j.ijscr.2025.112125

**Published:** 2025-10-28

**Authors:** Amol Gupta, Sanjeev Gianchandani, Swati Deshpande, Vasundhara Gopalan, Jhanwi Khurana, Arushi Kaul

**Affiliations:** Datta Meghe Institute of Higher Education and Research Deemed to be University, General Surgery, Wardha, 442001, Maharashtra, India

**Keywords:** Calcinosis cutis, Scrotum, Idiopathic, Dystrophic

## Abstract

**Introduction and importance:**

Calcinosis cutis, also known as calcification in soft tissue, is an uncommon benign condition that can be further classified into idiopathic, dystrophic, iatrogenic, metastatic, and calciphylaxis subtypes. The scrotum is one area where calcinosis cutis frequently occurs. During years or decades, the nodules gradually enlarge. The scrotal calcinosis cutis can be solitary or multiple, typically asymptomatic, hard, yellowish marble-like, polypoidal. Despite being benign, patients with this condition present late due to shyness or cancer anxiety, even when faced with sexual discomfort and infertility. In this article, we want to raise awareness to highlight benignity of this condition, various treatment options and good prognosis associated with the disease.

**Case presentation:**

A 38-year-old male presented with multiple painful, itchy scrotal swellings and infertility. Examination revealed scrotal nodules, absent left testis, and aspermia. Histopathology confirmed scrotal calcinosis. Total scrotectomy was performed with right testis repositioned in the thigh. Patient was referred for infertility management after successful surgical recovery.

**Clinical discussion:**

Idiopathic calcinosis cutis of the scrotum (ICCS) is a benign, slow-growing condition often presenting in adulthood with asymptomatic yellowish nodules. Diagnosis is confirmed histologically. Though idiopathic, it may involve dystrophic calcification of epidermoid cysts. Surgical excision with scrotal reconstruction is the preferred treatment, offering excellent cosmetic and curative outcomes.

**Conclusion:**

Being usually asymptomatic, ICCS is a benign disorder. It appears as a series of successive, different-sized nodules. A histological evaluation shows the calcified regions. One of two possible causes exists: idiopathic or dystrophic calcification of cysts. Excision is the preferred course of action.

## Highlights

• Definition and Rarity: Rare benign calcium deposition in scrotal skin, presenting as firm, asymptomatic nodules.
• Case Summary: 38-year-old male with multiple scrotal nodules for 10 years, aspermia, absent left testis, histology confirmed ICCS.
• Treatment: Total scrotectomy done, right testis shifted to thigh pocket, suction drain placed to avoid fluid buildup.
• Pathophysiology Debate: Cause debated—either idiopathic or from dystrophic calcification of ruptured epidermoid cysts.
• Diagnosis and Management: Histology confirms diagnosis; surgical excision with reconstruction prevents recurrence and ensures good results.

## 1 Introduction

Calcinosis cutis is the term used to describe abnormal calcium accumulation in the skin or subcutaneous tissue. Calcium does not accumulate in the skin and is always a harmful condition. All areas of the skin may be affected by calcification. The vulva, penis, or scrotum are examples of genital skin that might have idiopathic calcinosis. Lewinski initially identified idiopathic scrotal calcinosis, also known as idiopathic calcinosis cutis of the scrotum (ICCS), in 1883. It is a rare condition. The pathophysiology of scrotal calcinosis has been the subject of discussion recently, with some arguing that it is idiopathic. Lesions vary greatly in size and number and are typically asymptomatic. The characteristic of calcinosis cutis of the scrotum is usually slowly developing yellowish nodules with calcification and granulomatous inflammation resembling that of a foreign body.

## 2 Pathophysiology

The most frequent cause of calcinosis cutis is dystrophic calcification, which is linked to normal serum calcium and phosphorus levels. Systemic sclerosis, dermatomyositis, mixed connective tissue disease, or lupus are some of the underlying conditions that cause tissue destruction and calcification. Serum levels of calcium and phosphorus are aberrant in metastatic calcification, and deposition happens when the calcium phosphate product rises over 70. There is no underlying tissue injury or aberrant test results associated with idiopathic calcification. Scrotal calcinosis, subepidermal calcified nodules, and tumoral calcinosis are among them. When calcium or phosphate-containing agents are administered, calcium salts precipitate, which results in iatrogenic calcification. Dialysis and chronic renal failure are linked to calciphylaxis, which is characterised by the calcification of small and medium-sized arteries [1].

## 3 Case presentation

A 38-year-old male came with c/o multiple swellings on the scrotum since 10 years. Swelling covered the entire scrotal region. It was associated with pain, itching and powdery discharge. No history of any medical or surgical intervention in past. No history of any drug allergies. Patient is married since 8 years and did not have any child. This is not only a cosmetic problem but also a problem in the sexual life of the patient.

**Image 1:**
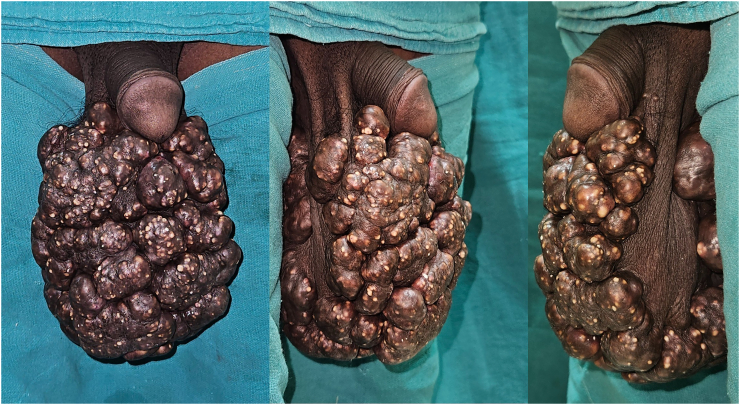
Clinical Presentation showing multiple nodular swelling.

On inspection: There were multiple nodular swelling covering the entire scrotum with normal penile structures, and no evidence of active bleeding or discharge (Image 1).

On palpation: Multiple swellings, which are hard in consistency and mobile in nature. The left-side testis was not palpable. The right-side testis was palpable.

**Image 2:**
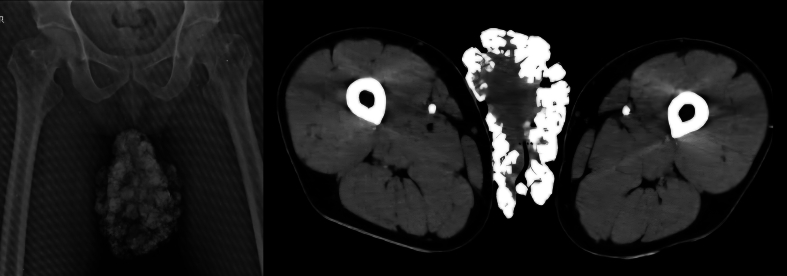
Radiological findings. 2a Xray, 2b CT scan (Multiple calcified lesions on scrotum).

Radiological imaging (Image 2) along with semen analysis was done which suggests absent left testis and aspermia. There is no correlation of infertility with calcinosis cutis. We also evaluated patient's spouse and she was found to be fertile.

**Image 3:**
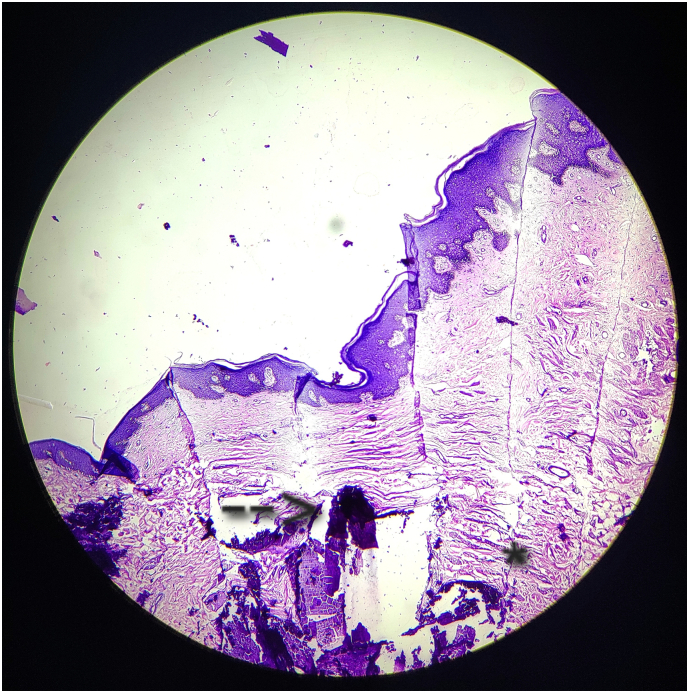
Histopathological image - Section show stratified squamous lining epithelium and multiple varying sized, granular, amorphous fractured calcium deposits within the dermis and subcutis surrounded with elastic fibroblast proliferation suggestive of calcinosis cutis.

**Image 4:**
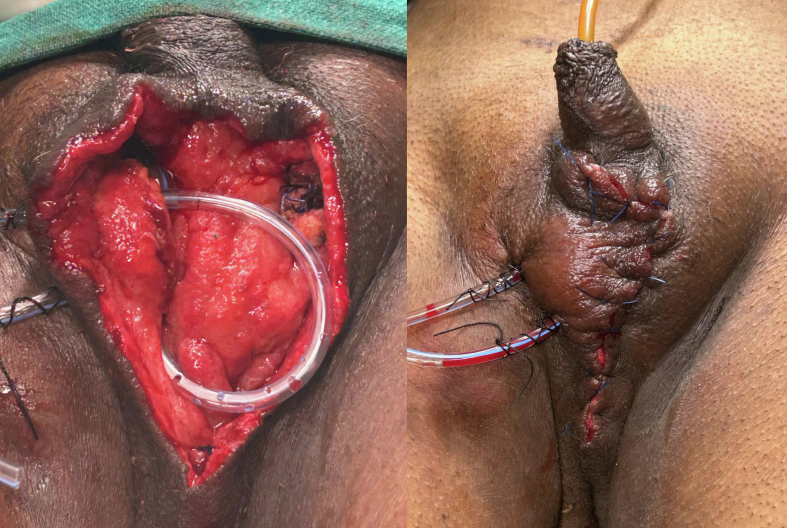
Intraoperative findings.

We excised one lesion from the scrotum and sent it for histopathological examination, which confirmed our diagnosis as calcinosis cutis of the scrotum due to absence of sebaceous lining (Image 3). We have performed total scrotectomy with right testis repositioned by creating a subcutaneous pocket 2-3 cm below to inguinal crease in anteromedial aspect of right thigh (Image 4) and close the skin incision using ethilon 3-0RC. We also kept a negative suction drain to prevent accumulation of any reactionary secretion in the cavity which was removed on post-operative day 5. There was minimal restrictions in mobility which eventually settled down within 15 days.

After complete recovery patient was advised to take urologist/endocrinologist opinion for the management of infertility. But patient was lost to follow up.

## 4 Discussion

Despite its early onset, individuals with ICCS often present in their third or fourth decade of life. It is a benign illness with few symptoms [2]. Delay in presentationtothe hospitalis due to its shyness and location [3]. Slow-growing, yellowish nodules in the scrotum that range in size from one to several are the disease's initial symptom. Itching may occasionally be connected to it, or the nodule may decompose and release chalky material. It is rare for the nodules to get infected [4].

The clinical and histological diagnosis is ICCS. The age of onset, size, number, and skin surface all point to ICCS, but histology is always used for confirmation. Steatocystoma multiform, angiokeratoma, lipomata, fibromata, and lymphangioma circumscriptum are among the potential diagnosis for a nodular lesion in the scrotum. Clinically, only calcified sebaceous cysts would be a challenge to differentiate from ICCS. Others can be easily disregarded [5]. Because the illness is idiopathic, its precise cause is unknown. According to several publications, ICCS is a misnomer, with the underlying cause being the calcification of the scrotal epidermoid or epidermal cysts.

Noel et al. studied 15 nodules from each patient in a case series of five patients who had many calcified scrotal nodules. They came to the conclusion that these nodules were calcified epidermoid cysts [6]. Calcified nodules were caused by calcification of hair follicular or epidermal cysts in another investigation including 20 patients with scrotal calcinosis [7].

The follicular cyst grows, followed by internal and external calcification, and ultimately the epithelial lining goes away. However, it is unclear what causes this calcium accumulation. Trauma may function as a trigger in specific situations [8]. By employing antikeratin monoclonal antibodies to identify keratin deposits within or surrounding the calcium aggregates, Wright et al. have previously refuted this notion. The lack of keratin buildup they discovered lends credence to the disease's idiopathic nature. Recent research by Yuyucu Karabulut et al. has further supported the dystrophic theory by demonstrating the presence of calcium granules and keratin fibers in the surrounding dermis [9].

In addition to the dystrophic calcification of eccrine glands, it is also the epidermoid cyst [10]. Additionally, dartos muscle is put out as a substitute Theory. Our case's histological results showed no indications of an epithelial lining. Multinucleated Giant cells were present in the calcifications [11].

It is necessary to rule out the possibility of calcification elsewhere in order to classify the illness as idiopathic. To identify the cause, a comprehensive biochemical and hormonal profile might be helpful. In the event that the illness is indeed idiopathic, the laboratory tests clearly fall within normal limits. It is not beneficial to use a single diagnostic method. With modest benefit, orders for X-rays, ultrasonography, and fine-needle aspiration cytology can be placed. Unless the nodules begin to discharge or become itchy, the treatment is purely cosmetic as the condition is benign and largely asymptomatic.

The preferred course of treatment is scrotal reconstruction after excision. It leaves behind a nice cosmetic outcome with little likelihood of recurrence. To stop a recurrence, even the smallest nodule needs to be removed.

Carbon dioxide laser light has been effective in treating small lesions and lesions on the digits [[12], [13], [14]].

## 5 Conclusions

Being usually asymptomatic, ICCS is a benign disorder. It appears as a series of successive, different-sized nodules. A histological evaluation shows the calcified regions. One of two possible causes exists: idiopathic or dystrophic calcification of cysts. Excision is the preferred course of action.

The work has been reported in line with the SCARE criteria [15]. Since patient was lost to follow-up, comment on post-op quality of life is not possible.

**Figure:**
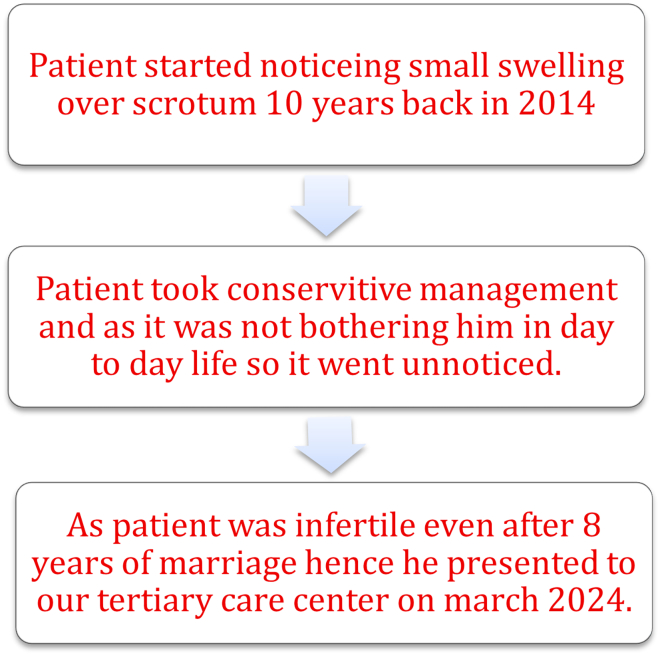

## Author contribution

AG: Conceptualization, design of the study, acquisition of data, drafting the article, revising it critically for important intellectual content, approval of the version to be submitted.

SG: Conceptualization, design of the study, acquisition of data, drafting the article, revising it critically for important intellectual content, approval of the version to be submitted.

SD: Analysis, drafting the article, revising it critically for important intellectual content, approval of the version to be submitted.

VG: Acquisition of data, analysis, revising it critically for important intellectual content, approval of the version to be submitted.

JK: Acquisition of data, analysis, revising it critically for important intellectual content, approval of the version to be submitted.

AK: Acquisition of data, analysis, revising it critically for important intellectual content, approval of the version to be submitted.

## Consent

Written informed consent was obtained from patient for publication and accompanying images.

## Ethical approval

Ethical approval is not required in our institution (Institutional Ethics Committee of the Medical University of Datta Meghe Institute of Higher education and research) for case reports and case series.

## Guarantor

Amol Gupta.

## Research registration number

Registration for research study is not applicable.

## Funding

The research did not receive any financial support.

## Conflict of interest statement

The authors declare no conflict of interest.
